# Networks as Biomarkers: Uses and Purposes

**DOI:** 10.3390/genes14020429

**Published:** 2023-02-08

**Authors:** Caterina Alfano, Lorenzo Farina, Manuela Petti

**Affiliations:** 1 Department of Experimental Medicine, Sapienza University of Rome, Viale Regina Elena, 324, 00161 Rome, Italy; 2 Department of Computer, Control and Management Engineering, Sapienza University of Rome, Via Ariosto, 25, 00185 Rome, Italy

**Keywords:** integrative biomarker, network analysis, precision medicine, biomarkers’ connectivity

## Abstract

Networks-based approaches are often used to analyze gene expression data or protein–protein interactions but are not usually applied to study the relationships between different biomarkers. Given the clinical need for more comprehensive and integrative biomarkers that can help to identify personalized therapies, the integration of biomarkers of different natures is an emerging trend in the literature. Network analysis can be used to analyze the relationships between different features of a disease; nodes can be disease-related phenotypes, gene expression, mutational events, protein quantification, imaging-derived features and more. Since different biomarkers can exert causal effects between them, describing such interrelationships can be used to better understand the underlying mechanisms of complex diseases. Networks as biomarkers are not yet commonly used, despite being proven to lead to interesting results. Here, we discuss in which ways they have been used to provide novel insights into disease susceptibility, disease development and severity.

## 1. Introduction

Biomarkers are commonly defined as measurable characteristics that can be used as indicators of normal biological processes, pathogenic processes or responses to an exposure or intervention; they can be of different nature, being derived from molecular, histologic, radiographic, or physiologic data [1].

Given the recent innovations and developments in molecular biology and biotechnology, molecular biomarkers have become increasingly investigated and proposed for patient stratification for both diagnosis and therapy response [2]. Indeed, recent advances in molecular biomarker-based diagnostics are redefining how diseases should be categorized and/or differentiated [3]. However, complex diseases are often caused by the interplay of a group of interacting molecules, rather than from the malfunction of an individual gene or protein [4]. While most genetic analysis approaches focus on individual genetic determinants and are therefore unlikely to characterize the network architecture of complex diseases comprehensively [5], the new drive toward precision medicine is increasing the need for more comprehensive disease biomarkers. However, considering the multitude of molecular interactions and their relationships with clinical indices is a challenging task [6]. Moreover, many diseases present multiple subtypes that manifest a similar pathological or physiological outcome and, to better understand them, it is necessary to analyze a variety of clinical, physiological, imaging, pathological, and biochemical features [7].

Network analysis has the potential to provide a holistic approach to the understanding of disease complexity, rather than focusing on individual components of disease. A network is a set of nodes and a set of edges between the nodes. Both nodes and edges can have additional attributes and the connections between nodes may or may not be directional (i.e., directed or undirected networks). In biological applications, nodes can represent different biological entities (e.g., genes, proteins, metabolites), while edges can model gene co-expression, physical protein–protein interactions and so on. Given their adaptability to different scenarios and their ability to model complex systems, network-based approaches have been applied extensively and successfully in biology and medicine. The most common uses include the analysis of gene expression data [8,9,10], computational disease gene prediction [11,12,13,14,15] or protein–protein interactions. However, network modelling and analysis have not been widely applied to the study of disease-related phenotypes [7] and in the definition of a disease biomarker based on the integration of all the relevant features characterizing patients. Modeling “networks as biomarkers” would thus allow to consider, at the same time, the different relevant features of a disease and their inter-relationships, giving a comprehensive view of the intricate underlying molecular mechanisms of a disease. This is not yet a common practice: many studies focus in fact on one category of biomarkers at a time, investigating the presence of a relation between two “views” (e.g., gene expression and clinical features) by means of pairwise correlation [16]. Including more layers of information would instead have a great impact on clinical practice. Therefore, even if not yet so common, this new paradigm is starting to spread, leading to its inclusion in studies of different medical fields and to the development of state projects on this specific topic [17].

The idea of studying the interactions between different biomarkers of the same disease is considered of great clinical impact, and some papers started considering networks of biomarkers as a single, comprehensive and integrative biomarker: this means considering the whole set of disease-relevant features and their interactions as a macro-biomarker to stratify patients based on its diagnostic and prognostic value. This is of particular interest in a dynamic network scenario, through which the changes through time could be measured and studied [18]. In fact, dynamic networks enable us to investigate how the interactions evolve over time, by having multiple instances of the same networks at different time points [19]. Oftentimes, molecular interactions are altered, not only because of major disorders or drug assumption, but also for physiological reasons (e.g., interactions between the genes involved in embryogenesis vary throughout development to ensure the correct development of the embryo) [20,21]. Sadly, most of these network-based analyses still tend to only consider genes or proteins as biomarkers, as shown by Wu et al. [4].

In this review, we will first highlight the benefits and challenges of integrating different biomarkers, to then focus our attention on papers whose purpose was to construct a network of relationships between biomarkers of different natures (e.g., gene expression, immune reactions, mutational events, age, BMI) and the methods they proposed. 

## 2. The Need for a Comprehensive and Integrative Biomarker

Medical practitioners have always been trying to “individualize” treatment strategies based on the patients’ characteristics and their acquired knowledge. Given the ongoing technological advancement, the information we can obtain about both patients and a disease is increasing in quantity as well as complexity. The sole concept of biomarkers has changed how we think about diseases and therapies. Biomarkers are commonly used to identify susceptibility to certain diseases and to predict prognosis and therapy response. Biomarker-driven patient stratification can provide clinicians accurate assessments of patient status, which in turn makes it possible to plan personalized treatments based on the information extracted from biomarker profiles [3]. However, the single-biomarker paradigm has flaws: a single biomarker may prove imperfect for the aim for which it has been proposed: for example, PD-L1 expression on tumor tissues has been shown to be an imperfect biomarker for immunotherapy response [22]. The more information we integrate, the more we can understand about both the disease and the patient. For example, studies focused on the analysis of molecular data often neglect to consider clinical information and its relationship with the molecular features. Understanding the interaction between clinical data and bioinformatic analysis is seen as the first and critical step towards the development of individualized therapy [23]. Wang and Liotta [23] specifically underline that “the simultaneous evaluation of clinical and basic research could improve medical care, care provision data, and data exploitation methods in disease therapy and algorithms for the analysis of such heterogeneous data sets”. This is why there is a strong advocacy for what is sometimes called “clinical bioinformatics” [23]. Clinical bioinformatics can be integrated into other typical methodologies that focus on metabolic and signaling pathways, biomarker discovery and development, computational biology, the various omics (such as genomics, proteomics, metabolomics, pharmacomics, transcriptomics), high-throughput image analysis, systems biology, etc. [23].

However, using clinical data is linked to some technical difficulties, such as the risk of privacy breaches for the patients and the lack of a standardized vocabulary for clinical informatics, which poses an obstacle in the development of automated systems. Nonetheless, the widespread adoption of the electronic medical record (EMR) system has generated large amounts of heterogeneous clinical data, both structured and unstructured. This allows comprehensive phenotypic–genotypic association studies to be conducted using the genotypes obtained from whole genome sequencing of a given cohort in combination with the phenome data of the same population, as available in the EMR database [17]. The American Medical Informatics Association (AMIA) [24] has also stressed that as a first point of action, we should aim at the creation of a database structure to unify clinical and genomic data, to allow the connection between biology information and patient health records. Similarly, the INBIOMEDvision is a two-year initiative funded by the European Commission 7th Framework Program of Information and Communication Technologies (ICT), with the aim of bridging the communities of bioinformatics and medical informatics [17].

A different perspective to the typical genomic approach can also be provided by including metabolomics data. In fact, it has been highlighted how a macromolecular, “bottom up” view of system activity cannot provide all the answers needed for precision approaches. Metabolic profiling instead offers a dynamic “top down” view of system activity and is a time dependent measurement well suited to test responses to drugs, environmental stimuli or disease. “This approach provides rich micromolecular data downstream of the genome and proteome, offering a genuine functional “snapshot” of system activity” [2].

Another similarly interesting point of view to look at a disease is the one offered by radiomics analyses. “Radiomics is an emerging field of study in which quantitative, high-throughput data are extracted, processed and analyzed to discover the associations with meaningful information. High-dimensional radiomic data provide insight into intra-regional heterogeneity by identifying sub-regions and reflecting the spatial complexity of a disease. Therefore, radiomics is an especially promising tool for personalized medicine in oncology, which requires a clear understanding of tumoral heterogeneity in individual patients” [25].

**Figure 1:**
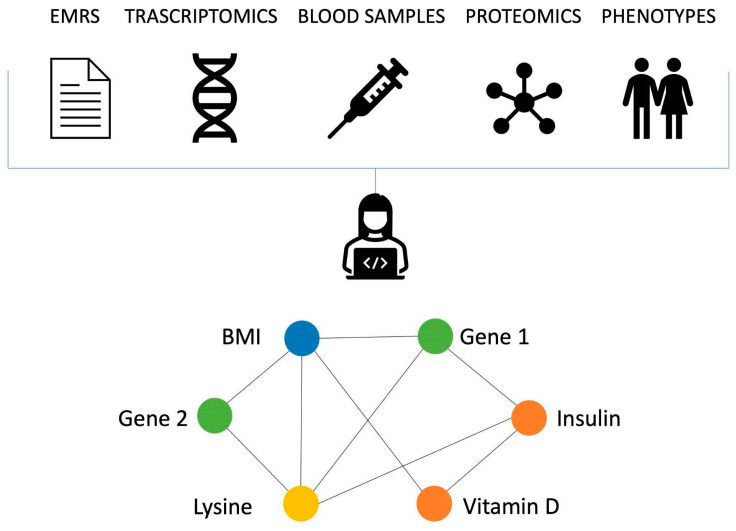
Networks of biomarkers showcase the underlying relationships between biomarkers of different natures. Data gathered from different sources (such as DNA sequencing, blood samples and electronic medical records) can be studied together as a whole system to shed light onto the molecular mechanism of a disease and help the diagnosis process. Here, biomarkers obtained by the same process are shown with the same colors. Biomarkers of different natures can be positively or negatively correlated.

There are clear benefits in integrating different types of data in terms of the information this can provide, and network-based approaches are a great tool to conduct such an analysis. Figure 1 shows as toy example of a network of this kind.

## 3. Networks as Biomarkers: State of the Art

Not many studies have solely focused on a network analysis of different biomarkers, so there is not a standard pipeline for such an analysis. Chu et al. [7] introduced an approach to infer networks of clinical biomarkers based on partial correlations. Working on chronic obstructive pulmonary disease (COPD), they focused on deconvoluting the known disease-related phenotypes and defining their relationships to one another and to specific genetic determinants. They reckoned that despite having a big potential in this field, network medicine approaches to complex diseases have focused on finding the relationships between diseases and the underlying cellular and molecular interaction network. Consequently, they defined their own network approach for the analysis of disease-related phenotypes. To infer their network, they used the Gaussian graphical model (GGM), which assumes that the variables have a Gaussian distribution and infers the connection between each pair of variables through partial correlations. Doing so, they measured the correlation between two variables while controlling for all other variables and computed the *p*-values associated with the partial correlation coefficients Finally, they also tested the differences between the networks of two groups of subjects by performing permutation tests.

The phenotypes they considered for the network analysis were 10 key quantitative COPD-related phenotypes based on clinical experts’ opinions. They were chosen to represent major disease-related components, and included imaging, physiology, exercise capacity, and exacerbations, as well as important demographic variables. To infer directionality, they also used the method proposed by Opgen-Rhein and Strimmer [12], which is based on the log ratios of standardized partial variances and leads to the identification of a “partially directed graph”.

They gathered their samples from the COPDGene Study [26] (a multi-center genetic and epidemiologic investigation to study COPD and other smoking-related lung diseases); the subjects who were missing data in any of the 10 quantitative variables were excluded, so their total population was made of 8141 subjects. Finally, they considered a second independent COPD population (which differed mostly in patients’ COPD severity and race and because only 8/10 variables were available) and compared the results between the two networks made from the two datasets, showing that they were very similar.

While discussing their results, they state that thanks to correlation-based networks, they were able to detect novel relationships between disease-related phenotypes that would not have been observed in a single-variable analysis. They identified phenotypes that had a key role in the network and found an intriguing switch in the direction of the relationship between some variables when comparing cases and control subjects. In the conclusion of their work, they also argued that this method can be applied in integrative analyses, including both phenotypic and genetic information. In particular, they tested it by examining how genetic perturbations affected the relationships between the biomarkers: in their example, they compared groups of subjects homozygous for risk and non-risk alleles at known GWAS SNPs and saw that few of the discovered interactions were different between homozygotes for alternative alleles of COPD GWAS regions near HHIP and FAM13A (two SNPs associated with COPD).

In a similar way, Park et al. [27] tried to understand at a biological level how regular exercise helps to prevent cardiovascular and metabolic diseases (CMD), such as diabetes and hypertension. To carry out this study, they used a very big cohort (17.000) of middle-aged subjects that took part in the Health Examinees Study of 2004; these subjects had completed questionaries about their exercise habits and were examined through both anthropometric measures and laboratory tests to collect information about 42 biomarkers. Among other statistical analyses, they created a network based on the significant differential correlations between the exercise and non-exercise groups, considering men and women separately. To do so, they computed partial correlations adjusted for age among the considered biomarkers. They then identified significant differential correlations between the exercise and non-exercise groups and visualized the ones with correlation coefficients greater than 0.1. Doing so, they found that “body composition-related biomarkers were likely to play major roles in men, while obesity-related biomarkers seemed to be key factors in women”. However, they also state that the biomarkers they considered might not be enough and that “including metabolomics or microbiome data would allow to show more comprehensive relationships” and to better identify the biomarkers that may explain the health benefits of regular exercise on preventing chronic diseases.

A different approach has been used by Huang et al. [28] who decided to study the interconnectivity of biomarkers individually related to the risk of type 2 diabetes, as well as the potential changes in the biomarker correlation network during diabetes development. In fact, although many studies have examined different biomarkers separately, it remains unclear, at the system level, how some biomarkers may interact with others belonging to/related to different biologic pathways and contribute to diabetes development.

The authors of this paper focused specifically on “27 plasma biomarkers representing glucose metabolism, inflammation, adipokines, endothelial dysfunction, IGF axis, and iron store (plus age and BMI) at blood collection from an existing case–control study nested in the Nurses’ Health Study (NHS), including 1303 incident diabetes case subjects and 1627 healthy women”. The aim of their analysis was to construct a network with biomarker relationships that were statistically different between case and non-case subjects, so they computed pairwise Spearman correlations between them, keeping case and non-case subjects separated. Then, they evaluated the difference in correlation between the two groups and assessed the statistical significance of the difference using permutation tests that randomly assigned the case and non-case status and calculated the correlations between the reassigned groups. They repeated this process 1000 times and obtained the distribution of the correlation differences, on which a standardized correlation difference was calculated and selection criteria were determined to choose which correlations to evaluate in the network analysis. They then performed a sensitivity analysis to check both the impact of changing the selection criteria and to test the impact of the age and BMI variables that they had added to the plasma biomarker. Finally, to understand the biomarker network’s patterns in the pathogenesis of diabetes, they further evaluated the network structure separately among diabetes case subjects diagnosed in different periods of time relative to the blood collection with the non-case subjects.

Thanks to this approach, they were able to find significant differences between diabetes case and non-case subjects, while also noticing that the biomarker correlation structure was disturbed many years before the clinical diagnosis of diabetes. They were also able to highlight the “central role of the leptin system on multiple biologic systems that may act synergistically in the pathogenesis of diabetes” as well as “the decade-long persistent dysregulation between insulin and HbA1c throughout the development of diabetes”.

A similar approach was carried out by Nishihara et al. [6], who hypothesized that the correlation network structures and hub biomarkers might differ between proximal and distal colorectal cancer, because they show a distinct set of molecular pathological signatures. From the colorectal cancer databases of the Nurses’ Health Study (NHS) and the Health Professionals Follow-up Study (HPFS) they were able to gather a set of patients and control cases equally split and matched by age and sex. The biomarkers used for this analysis included somatic oncogenic mutations, epigenetic features, protein expression levels, and host immune reactions in colorectal carcinoma, for a total of 54 biomarkers. On these biomarkers (which were either continuous, ordinal or binary), they performed pairwise complete Spearman correlations and selected the statistically significant ones, based on the Bonferroni correction. These correlations were used to construct two networks, one for distal colorectal cancer and one for proximal colorectal cancer. They then compared the two networks and used Kolmogorov–Smirnov test to evaluate the distance between the cumulative degree distributions of the proximal colon cancer network and the distal colorectal cancer network. Hubs of the network were defined as nodes with a degree centrality greater than the 80th percentile, based on the overall colorectal cancer network, and for each node, a clustering coefficient was also computed, as well an average of clustering coefficients for the networks themselves. To identify which biomarkers had higher connectivity in the proximal colon cancer network with respect to the distal colorectal cancer network, they then computed Cook’s distance for each highly connected node. Finally, they performed a series of permutation and sensitivity analyses to assess the robustness of their results.

They found that “biomarkers in proximal colon cancer possessed higher connectivity while those in distal colorectal cancer tended to be independent from each other”. They also saw that in proximal colon cancer only, MSI-high (microsatellite instability) and BRAF mutation occurred in relation to many other tumor features and realized therefore that the role of MSI differs by tumor anatomic location, stressing the importance of considering multiple correlated pathways for therapeutic targets.

As already stated, metabolomics has proven to be a valuable emerging tool to study the changes in phenotype caused by disease, by focusing on low-molecular-weight compounds, which are the final downstream products of gene expression. With the rapid growth of mass spectrometry (MS), it has become increasingly popular to evaluate metabolic disorders [29]. There are many approaches to extract useful information from metabolomics data, but network theory has proven to be particularly suitable for this purpose, since the variety of organic metabolites and the wide range of their concentrations always made it hard for researchers to understand metabolomics experimental results [30].

Wang et al. [29], for example, focused on exploring the variances of metabolic profiles for the early diagnosis and treatment of hypertension by analyzing plasma samples from twenty young hypertensive men and their age-matched healthy controls. They performed principal component analysis (PCA) for group discrimination and identified altered metabolites between groups using the non-parametric Mann–Whitney–Wilcoxon test. The adjusted *p*-values with less than a desired FDR (5%) were considered significant. Then, to provide a biological interpretation, pathway analyses were performed on the 27 significantly altered metabolites and were found to be enriched for the biological module of amino acids biosynthesis. Then, using all 70 available metabolites, they constructed a network based on Pearson correlation coefficient; to construct the correlation network, they determined an appropriate threshold (in the range 0.4–1) by evaluating its effect on the average degree, network diameter and size of the largest connected components. They then constructed the correlation network by using metabolic data of both the patient and control groups and computed a series of statistical characteristics, including degree, average path length, diameter, and clustering coefficient. For each node, three centrality indices (degree centrality, betweenness centrality and closeness centrality) were also assessed. The four most connected nodes were designated hubs of the network and were found to be significantly different between young hypertensive men and healthy men. At this point, they also constructed a network focused on just 20 altered metabolites, which showed the important role played by some amino acids. The authors stated, however, that comparing two separate networks, one for hypertensive men and one for healthy controls, would complement the results of their study.

Having also found an interesting role of fumarase, two years later a second study was carried out by some of the authors to delve into this result. To better understand the metabolism profiles induced by fumarase insufficiency, they again used a “GC–MS based metabolomics platform coupled with a network approach to analyze fumarase insufficient human umbilical vein endothelial cells (HUVEC) and negative controls” [30]. The same analysis pipeline was carried out on 62 metabolites (including organic acids, amino acids, fatty acids, sugars and nucleic acids), 24 of which were significantly altered in the fumarase-insufficient HUVEC. Among them, about seven metabolic pathways were identified as significantly altered, and they were mainly enriched with the biological module of amino acids metabolism. Differently from their previous study, after constructing the network and computing the above-mentioned metrics, they ranked the metabolites according to the PageRank algorithm based on the three centrality indices. As a result, they identified the hubs of the network, which were again also altered metabolites.

Lin et al. [31] focused instead on analyzing the variances of plasma metabolites to identify potential therapeutic targets for diabetes. They used 184 plasma samples of Chinese males, aged between 40 and 60 with similar BMI and with no family history of diabetes and smoking. On these data, they performed a binary logistic regression to remove the interference of confounding factors on diabetes. They then used the SIMCA-P software to perform supervised partial least squares discriminant analysis (PLS-DA) to examine the separation of data between different groups and identified differential metabolites between the two groups using the T test. The differential metabolites were used for both enrichment and pathway analysis. To construct the network, correlations between metabolites were measured with a Pearson correlation coefficient, using 0.5 as threshold. The Fruchterman–Reingold algorithm was then used to analyze the nodes and compute centralities measures, later used to identify the network’s hubs.

They found that “protein biosynthesis” and “urea cycle” were among the most enriched pathways of the altered metabolites, which were mainly involved in amino acid and glycerolipid metabolism. Thanks to the network analysis, they were able to characterize the metabolic profiles of plasma of diabetic patients with respect to non-diabetic controls, to find the pathways most affected by diabetes and to notice that glycerol, alanine and serine were “vital metabolites of diabetes”.

Given the successful results of network approaches in metabolomics analyses, more integrative studies were carried out since. Li et al. [32], for example, worked on a comprehensive study, that included more than just metabolomic data. In fact, they decided to integrate data from the gut microbiota, with blood parameters and urine metabolites of carotid atherosclerosis treatment-naive individuals. Their selection shows a wide range of metabolic disease phenotypes and is due to their belief that comprehensive analyses of multi-omics data can provide insights into interactions between different biological layers, even if they concern distinct domains. To integrate all their data, they performed three rounds of pairwise Spearman correlations, retaining triangular correlations for adjusted *p* of < 0.05. Moreover, they only considered correlations with rho greater or equal than three. They then selected only inter-omic correlations to perform a community analysis with the Girvan and Newman algorithm.

The authors also predicted clinical parameters with a 5-repeated 10-fold down-sampling cross-validation random forest model and checked its accuracy by computing a Spearman correlation between the predicted value and the measured value. They also implemented a non-carotid atherosclerosis versus carotid atherosclerosis classification.

As a result of their analysis, they found that variations in one of the three considered systems correlated with changes in the other two, and that “candidate gut microbial biomarkers and urine metabolite features were covaried with distinct clinical phenotypes”. In fact, the inferred network presented two main clusters: one large gut microbe–urine metabolite close-knit cluster and one triangular cluster composed of a gut microbe–blood–urine network. This demonstrated the existence of a close inter-system crosstalk, especially between the gut microbiome and the urine metabolome.

Table 1 collects a summary of the studies here reported, with details about the considered nodes and the chosen procedure to infer edges.

## 4. Discussion

The literature on this topic confirms not only that the use of networks as biomarkers has been theorized as a helpful tool, but that it has also been successfully tested on different diseases. However, a standard analysis pipeline does not emerge clearly from the discussed studies: a stronger incidence is seen with Pearson or Spearman correlations; however, depending on the data and the purpose, others consider it more appropriate to use partial-correlations-based methods. In fact, if by using Spearman or Pearson we can obtain a system-level representation and go beyond the classical approach, by computing partial correlations, we are considering the other features even during the estimation of the nodes’ relationships.

The studies that suggest using a network of biomarkers have the aim of investigating the relationships between different disease relevant features: in fact, the first to introduce the definition of “biomarker network” [6] suggested it as a method for analyzing two subtypes of colorectal cancer and indeed found that the role of MSI differs by tumor anatomic location. The following studies performed similar analyses, having mostly data exploratory purposes. However, this approach does not only allow the links between different features to be understood, but it could also be of use in the definition of a network as a comprehensive and integrative biomarker.

## 5. Conclusions

Network-based approaches have been proven to be extremely helpful to put together and analyze in a comprehensive way the complex system of molecular interactions underlying human diseases. Such an approach has also been successfully applied to studies focused on non-genetic biomarkers or biomarkers of different natures at the same time. Despite these successful results, and the documented need for the integration of features belonging to more domains, this approach is not yet a common practice.

In this review, we highlighted the potential of network approaches when studying disease biomarkers and presented an accurate summary of the methods and strategies that have currently been tested in the literature. Each of these studies stressed the importance of such an innovative approach in reaching the results they obtained at the end of their process, proving that there are many useful possibilities from carrying out analyses of this kind. Such methodologies would be particularly useful in a precision medicine scenario, by producing results that could allow a more defined stratification of the patients, as well as having a potential impact on early detection and prognosis.

## Figures and Tables

**Table 1:** Literature overview: methodologies to construct networks of biomarkers.

Reference	Data	Edge Definition
Huang, T. et al., 2019 [28]	27 plasma biomarkers (glucose metabolism, inflammation, adipokines, endothelial dysfunction, IGF axis and iron store), age, BMI	Spearman correlation
Nishihara, R. et al., 2017 [6]	54 biomarkers (major mutational events, microsatellite instability, epigenetic features, protein expression status, immune reactions)	Spearman correlation
Chu, JH. et al., 2014 [7]	10 COPD-related phenotypes (FEV1, Emphysema, Emphysema Distribution, Gas Trapping, Airway Wall Area, Exacerbation frequency, Six-minute walk distance, BMI, Age, Pack years)	Partial correlations
Wang, L. et al., 2014 2/6/2023 9:07:00 PM	70 metabolites from plasma samples	Pearson correlation
Hou, E. et al., 2018 [30]	62 metabolites	Pearson correlation
Lin, W. et al., 2020 [31]	15 metabolites from plasma samples (nine amino acids and four fatty acids and glycerol)	Pearson correlation
Li, R.J. et al., 2021 [32]	645 Metagenomic species, 124 blood biomarkers, 1042 urine metabolite groups	Spearman correlation
Park, J. et al., 2021 [27]	42 biomarkers (anthropometric measures and laboratory tests)	Differential partial correlations

## Data Availability

No new data were created or analyzed in this study. Data sharing is not applicable to this article.
